# Preliminary design of a new degradable medical device to prevent the formation and recurrence of intrauterine adhesions

**DOI:** 10.1038/s42003-019-0447-x

**Published:** 2019-05-22

**Authors:** Salome Leprince, Stéphanie Huberlant, Lucie Allegre, Sophie Warembourg, Isabelle Leteuff, Audrey Bethry, Cedric Paniagua, Hubert Taillades, Renaud De Tayrac, Jean Coudane, Vincent Letouzey, Xavier Garric

**Affiliations:** 1 Institut des Biomolécules Max Mousseron (IBMM), UMR 5247, CNRS, Université Montpellier, ENSCM, Montpellier, 34093 France; 2 Department of Gynecology and Obstetrics, University Hospital, Nîmes, 30900 France; 3 Experimental Department, University of Montpellier, Montpellier, 34000 France

**Keywords:** Translational research, Experimental models of disease

## Abstract

Intrauterine adhesions lead to partial or complete obliteration of the uterine cavity and have life-changing consequences for women. The leading cause of adhesions is believed to be loss of stroma resulting from trauma to the endometrium after surgery. Adhesions are formed when lost stroma is replaced by fibrous tissue that join the uterine walls. Few effective intrauterine anti-adhesion barriers for gynecological surgery exist. We designed a degradable anti-adhesion medical device prototype to prevent adhesion formation and recurrence and restore uterine morphology. We focused on ideal degradation time for complete uterine re-epithelialization for optimal anti-adhesion effect and clinical usability. We developed a triblock copolymer prototype [poly(lactide) combined with high molecular mass poly(ethylene oxide)]. Comparative pre-clinical studies demonstrated in vivo anti-adhesion efficacy. Ease of introduction and optimal deployment in a human uterus confirmed clinical usability. This article provides preliminary data to develop an intrauterine medical device and conduct a clinical trial.

## Abstract (web summary)

Salome Leprince, Stephanie Huberlant et al. report the design of a degradable anti-adhesion intrauterine device that prevents formation of intrauterine adhesions following trauma or surgery. They show its efficacy in rats and its usability in the human uterus.

## Introduction

Intrauterine adhesions (IUAs) lead to a partial or complete obliteration of the uterine cavity and have important life-changing consequences for women because they provoke pain, menstrual aberrations have a debilitating impact on reproductive function^1–3^. IUAs result from trauma to the endometrium that occur after a cesarean section, spontaneous miscarriage, and intrauterine surgical procedures (polyp removal 3.6%^4^, resection of uterine septa 6.7%^4^, myomectomy 21.6%^5^, and abortion evacuation curettage 37.6%^6^) that damages the endometrial stroma. Loss of stroma is believed to be the leading cause of IUAs, which are formed when lost stroma is replaced by fibrous tissue that join the uterine walls. The resulting histological and morphological modifications of the uterine cavity cause acute pelvic pain, chronic discomfort, and mechanical infertility^7–10^.

The gold standard for treating IUAs is hysteroscopy adhesiolysis, a procedure that cuts the fibrous bridges to restore the uterine morphology, prevent recurrence, facilitate endometrial regeneration, and restore reproductive functions^3,11–13^. Rates of recurrence after surgery, however, are high for moderate (23%) and severe (62%) adhesions^14–17^. Alternative strategies to more effectively prevent the formation and recurrence of histological lesions in the uterine cavity have recently been proposed, and anti-adhesion barriers to prevent or limit the joining of uterine walls is widely considered to be the most effective^18^.

For lack of a better alternative, gynecologists treat intrauterine adhesions with resorbable barriers specifically developed for abdominal adhesions^19^.

Gynecological surgeons report that the design of these barriers make them difficult to insert via the vaginal route because they require dilation of the cervix, and the treatment often results in inadequate anti-adhesion effects for moderate to severe IUAs. Inadequate anti-adhesion effects are likely owing to elimination before complete uterine re-epithelialization can occur.

Complete re-epithelialization of the uterine cavity after trauma to the endometrium is crucial for effectively treating IUAs because it prevents the joining of endometrial walls.

The objective of our study was to develop an intrauterine medical device prototype designed specifically for IUAs. Specific objectives were to obtain adequate anti-adhesion effects and to develop a device that could be directly inserted after a surgical procedure via the vaginal route without requiring dilation of the cervix.

We developed a poly(lactide) copolymer film prototype designed to spread quickly over the entire uterine wall and degrade within the time frame considered sufficient for re-epithelialization to occur.

Degradation, swelling/deployment, and anti-adhesion properties were considered important in the design of the biomaterial used to create this prototype. Polylactides (PLAs) were used to design our medical device, as they are widely used for biomedical applications, biocompatible, and their rate of degradation can easily be modulated. PLAs, however, are rigid, cannot swell in an aqueous medium, and cannot limit cell adhesion and proliferation. To obtain adequate swelling properties and a degradation rate for optimal ant-adhesion effects, we combined a hydrophilic polymer with a high molecular mass^20^, a poly(ethylene oxide) (PEO), and a poly(lactide), resulting in a triblock copolymer PLA-PEO-PLA.

Anti-adhesion effect is defined as the absence of fibrous tissue along with complete re-epithelialization. The time frame the medical device should remain fully formed in the uterine cavity was estimated according to the number of days needed for complete re-epithelialization to occur in menstrual desquamation (5–6 days)^21,22^, and the number of days the device could remain within the uterine cavity without interfering with fertilization (15 days). We hypothesized that 15 days would be sufficient in severe cases of loss of stroma without interfering with pregnancy outcomes.

In this study, our design strategy took into account ideal degradation time for complete uterine re-epithelialization to allow for optimal anti-adhesion effect, and clinical usability for gynecological surgery. We organized this study into five steps: synthesis and characterization of copolymers; in vitro evaluation of copolymers swelling, degradation and anti-adhesion effect; in vivo evaluation of copolymer anti-adhesion effect and degradation in a rat model of sidewall defect–cecum abrasion and selection of the optimal copolymer; in vivo efficacy of the optimal copolymer for preventing intrauterine adhesions; and ex vivo in utero deployment of the optimal copolymer prototype.

We developed a degradable triblock copolymer prototype based on poly(lactide) and high molecular mass poly(ethylene oxide). Comparative pre-clinical studies in rats demonstrated in vivo anti-adhesion efficacy compared with current anti-adhesion products. Ease of introduction and optimal deployment in a human uterus confirmed its usability in a clinical setting. This study provided important preliminary data for the development of an anti-adhesion intrauterine device that could prevent IUAs and improve rates of fertility.

## Results

### Synthesis and characterization of copolymers

To develop degradable copolymers suitable for gynecological surgery, we copolymerized biocompatible thermoplastic materials that combined PLA segments with high molecular mass PEO. The advantage of this design is that lactic acid to ethylene oxide (LA/EO) ratios can be modulated. Several copolymers with different degradation rates, swelling properties, and anti-adhesion effects were synthesized and evaluated.

**Fig. 1:**
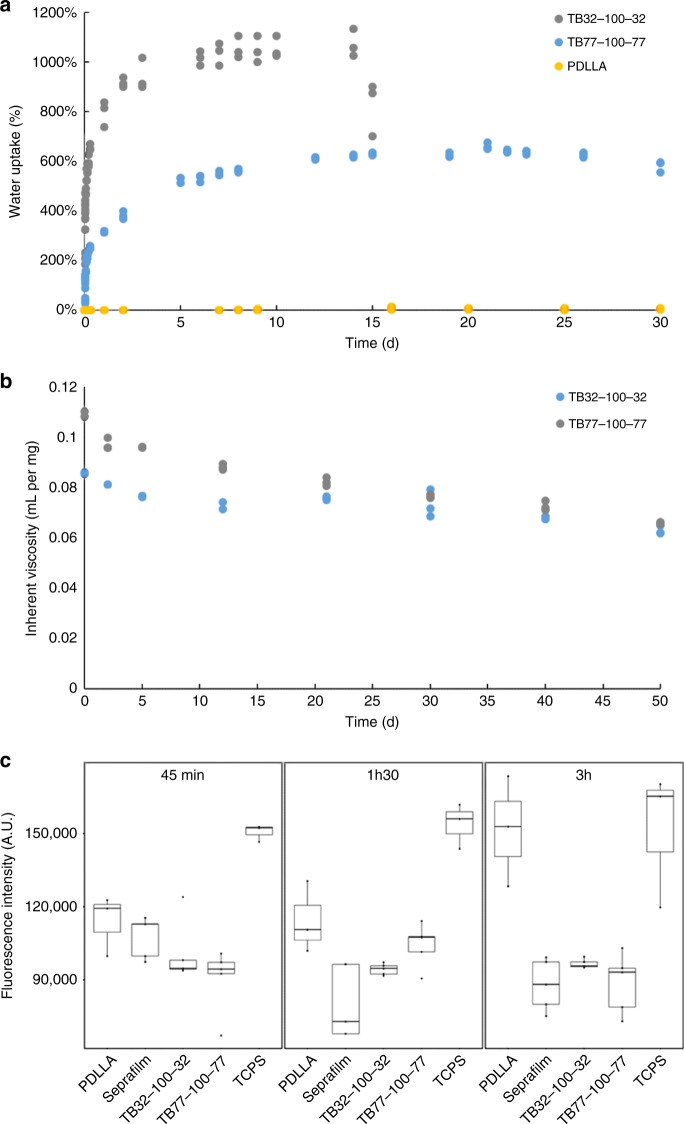
Water uptake, in vitro degradation and in vitro cell adhesion of copolymer films. **a** Water uptake (%) of triblock films and PDLLA in saline solution (initial pH 7.4) at 37 °C (*n* = 3 independent samples). **b** Inherent viscosity during in vitro degradation after immersion in saline solution (initial pH 7.4; 37 °C). **c** Adhesion of the NCTC 929 cells after 45 min, 1H30 and 3H on copolymer films, PDLLA film, Seprafilm®, and control group (TCPS). All data points and standard deviations are the result of *n* = 3 independent samples

PDLLA and two triblock copolymers (TB32-100-32 and TB77-100-77) were synthesized then characterized by nuclear magnetic resonance (^1^H-NMR), capillary viscosimeter, size exclusion chromatography (SEC), and differentieal scanning calorimetry (DSC) (Fig. 1). Molecular masses of PDLLA were determined by SEC. SEC analyses were also conducted for the triblock copolymers, but gave molecular mass values 10 × lower than those calculated by NMR. This difference was likely owing to inaccurate measurements caused by SEC calibration, which used polystyrene standards^23,24^. The amphiphilic nature of these copolymers could lead to inaccurate molecular masses owing to changes in hydrodynamic volume. To increase accuracy, molecular masses were calculated from the ^1^H-NMR integration ratio of the peak at 1.58 ppm (CH_3_ group in PLA block) and the peak at 3.6 ppm (CH_2_ group in PEO block). Final LA/EO molar ratios were also lower than initial ratios owing to incomplete lactide conversion during polymerization (Table 1). Absence of PLA homopolymers formed during the polymerization was then confirmed using diffusion-ordered spectroscopy.

**Table 1:** Characterizations of PDLLA polymer and PDLLA-PEO-PDLLA triblock copolymers

Polymer	Molecular mass of PEO (kg per mol)	Molecular mass of PDLLA (kg per mol)	Initial LA/EO ratio	Final LA/EO molar ratio^a^	Inherent viscosity^b^ (mL per mg)	Thermal properties^c^ *T*g/*T*m (°C)
PDLLA	0	101^d^	N.A.	N.A.	0.058	46/N.A.
TB32-100-32	100	64^a^	0.6	0.4	0.086	29/54
TB77-100-77	100	155^a^	1.2	0.9	0.109	42/56

^a^ Determined from the ^1^H-NMR integration ratio of the peak at 1.58 ppm (CH_3_ group in PLA block) and the peak at 3.6 ppm (CH_2_ group in PEO block)

^b^ Determined by capillary viscosimeter

^c^ Determined by differential scanning calorimetry

^d^ Determined by size exclusion chromatography

Characterization of the copolymers via capillary viscometer showed that inherent viscosity increased with molecular mass. DSC measurements of triblock copolymers showed an increase of the glass transition temperature (*T*g*)* as molecular mass increased. These results show that triblock polymers with a central block of high molecular mass PEO induces a decrease of *T*g compared with PDLLA.

### In vitro evaluation of copolymers

*Swelling properties*: Swelling properties, in vitro degradation and cell adhesion abilities were evaluated in TB32-100-32 and TB77-100-77. Water uptake was compared with PDLLA to validate using PEO to increase hydrophilicity (Fig. 1a). PDLLA films showed poor water uptake, whereas triblock films had increased after 1 hour for TB32-100-32 (500%) and TB77-100-77 (200%) with a continued increase over 10 days. Triblock film thickness also increased from ~ 600 to 1200 µm in 24 h.

This data demonstrate that triblock films increase in water uptake with decreasing LA/EO ratio. Water uptake also reached a plateau at day 14 for TB32-100-32 (1000%) and at day 22 for TB77-100-77 (600%). Degradation of PDLLA blocks and the solubilization of PDLLA oligomers could explain the decrease of water uptake observed after the plateau for TB32-100-32.

*Degradation*: In vitro degradation was measured through changes in inherent viscosity of the copolymers and inherent viscosity decreased progressively over 50 days, suggesting that molecular masses decreased owing to hydrolytic degradation of PDLLA blocks (Fig. 1b). After 50 days, copolymers were fragmented then solubilized in the degradation solution.

As the degradation rate of poly(lactid acid) based polymers increases in vivo^25^, we anticipated that the in vivo degradation time of the copolymers would be < 50 days.

*Cell adhesion effect*: Anti-adhesion properties were evaluated by deposing NCTC-Clone 929 cells on copolymer films, Seprafilm®, PDLLA film, and tissue culture polystyrene (TCPS) (cell culture control group) (Fig. 1c). Seprafilm® was chosen as a second control because of its insolubility in cell culture medium and its anti-adhesion properties^26^.

After 45 min, 1H30 and 3H, cells adhered less on TB32-100-32 compared with PDLLA and TCPS. We obtained the same result for TB77-100-77 after 45 min and 3H (95% confidence). At 1H30 the number of cells was not different from the number of cells on PDLLA films owing to the high standard deviation of the values.

The number of cells that adhered to TB32-100-32 and TB77-100-77 was equivalent to the number of cells that adhered to Seprafilm® after 3H of incubation.

Adhesions result from a dysfunction of the mechanism involved in fibrinolysis, which leads to an imbalance between fibrin formation and degradation^27,28^. In dysfunction of the fibrin degradation process, the fibrin matrix is colonized by fibroblasts. Limiting the adhesion of fibroblasts was, therefore, an important consideration in the design of this anti-adhesion barrier. The results of this study demonstrates that adding PEO in poly(lactic)-based polymers significantly reduces cell adhesion and is just as effective as commercially anti-adhesion barriers (Seprafilm®).

### In vivo evaluation of copolymers

In vitro experiments demonstrated that both copolymers films could meet clinical requirements in terms of degradation rate, anti-adhesion effect and swelling properties. It was also necessary to evaluate degradation rate and anti-adhesion effect in vivo. A well-known rat model of sidewall defect and bowel abrasion^29–32^ was used to evaluate the adhesion prevention efficacy of copolymers. This model also allows the collection of degradation products without risk of natural elimination and the evaluation of the degradation rate.

**Fig. 2:**
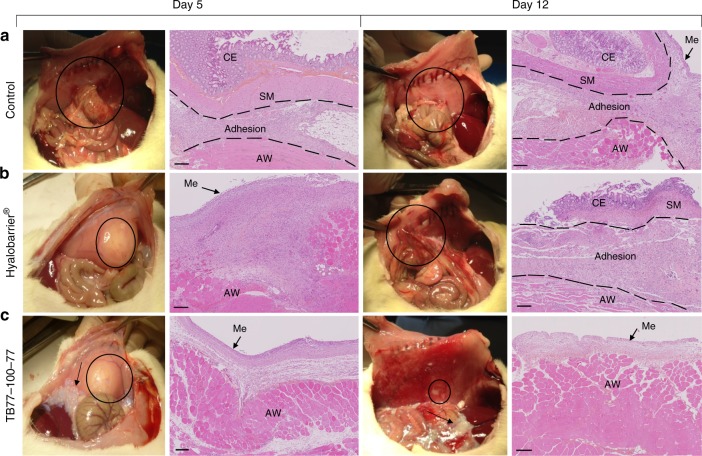
Prevention of peritoneal adhesions. Macroscopic images and histological examination (HES staining) of the wound site in rats 5 and 12 days after surgical trauma. **a** Control group: without treatment. **b** Group treated by instillation of Hyalobarrier gel. **c**. Group treated by implantation of TB77-100-77 films. In macroscopic images, circles indicate the site of peritoneal defect and black arrows indicate residues of TB77-100-77. For histological examination, Me: Mesothelial cells; CE: Cecal mucosa; SM: Visceral Smooth Muscle; AW: Abdominal Wall. Scale bars: 200 µm

*Anti-adhesion effect in peritoneal adhesion prevention*: TB32-100-32, TB77-100-77, and Hyalobarrier® (anti-adhesion barrier currently in use^19,33^) were evaluated for their ability to prevent peritoneal adhesions and compared with a surgical control group. Adhesion scoring (Table 2) was conducted at 5 and 12 days. Results are shown in Table 3 and macroscopic images and histological observations are presented in Fig. 2.

**Table 2:** Peritoneal adhesion: scoring system^34^

*Extent*	
No adhesion	0
1–25% of the cecum-peritoneal defect involved	1
26–50% of the cecum-peritoneal defect involved	2
51–75% of the cecum-peritoneal defect involved	3
76–100% of the cecum-peritoneal defect involved	4
*Severity*	
No adhesion	0
Filmy avascular	1
Vascular	2
Total attachment of cecum with peritoneal defect	3
*Degree*	
No adhesion	0
Require gentle traction to be freed	1
Require moderate traction to be freed	2
Require sharp dissection or not dissectible	3
Without damage to adherent organs	
Total adhesion score	0–10

**Table 3:** Incidence of peritoneal adhesions and postsurgical adhesions score per treatment group on postoperative day 5 and 12

Postoperative day	Category	Control group	Hyalobarrier® group	TB32-100-32 group	TB77-100-77 group
Day 5 (*n* = 6)	Incidence (%)	6 (100%)	0	3 (50%)	0
	Extent	4.00 ± 0.00	0	1.00 ± 1.55	0
	Severity	3.00 ± 0.00	0	1.17 ± 1.33	0
	Degree	2.66 ± 0.52	0	0.83 ± 1.17	0
	Total score	9,67 ± 0.52	0	3.00 ± 3.52	0
Day 12 (*n* = 6)	Incidence (%)	5 (83%)	3 (50%)	3 (50%)	0
	Extent	3.00 ± 0.00	1.67 ± 1.97	1.00 ± 1.09	0
	Severity	2.30 ± 0.00	1.33 ± 1.50	1.33 ± 1.50	0
	Degree	2.00 ± 0.00	1.50 ± 1.64	1.17 ± 1.47	0
	Total score	7.33 ± 3.88	4.50 ± 5.05	3.50 ± 3.99	0

The high incidence of peritoneal adhesions (83–100%) in the control indicates that our animal model is an effective means to evaluate anti-adhesion effects of our copolymers.

In the Hyalobarrier group, no adhesions were observed at day 5. At day 12, adhesions were observed with no significant reduction in incidence compared with the control (*p* = 0.5455). Adhesions were observed in the TB32-100-32 group at days 5 and 12 with no significant reduction in incidence compared to the control (*p* = 0.1818 and *p* = 0.5455, respectively). No adhesions were observed at days 5 and 12 in the TB77-100-77 group, indicating that postoperative adhesion formation was significantly lower compared with the control (*p* < 0.05).

At day 5, total adhesion score of the control group was 9.67 compared with the treated groups (Hyalobarrier = 0, TB32-100-32 = 3, and TB77-100-77 = 0). The difference between adhesion scores was statistically significant (*p* < 0.05). At day 12, total adhesion score of the control group was 7.33 compared with the treated groups (Hyalobarrier = 4.5, TB32-100-32 = 3.5 and TB77-100-77 = 0). The difference between adhesion scores of the control compared with the TB77-100-77 group was statistically significant (*p* < 0.05). There was no statistical significance between the control and Hyalobarrier and TB32-100-32 groups.

Adhesions observed in the TB32-100-32 group had notable scores in terms of extent, severity and degree at 5 and 12 days and were likely owing to rapid degradation of the copolymer (absence of residual degradation products at 5 days). The Hyalobarrier group did not develop adhesions after 5 days, but developed severe and extended adhesions at day 12 owing most likely to rapid elimination. In contrast, the TB77-100-77 group did not develop any adhesions at day 5 and 12, suggesting a more-effective anti-adhesion capability owing most likely to moderate degradation.

Histological examination was conducted for the control, Hyalobarrier, and TB77-100-77 groups (Fig. 2). Samples from the control group at days 5 and 12 showed adhesions between the abdominal wall and the cecum with large amounts of inflammatory cells, fibroblasts, and collagen fibers (Fig. 2a) indicating severe adhesions.

Tissues from the Hyalobarrier group at day 5 showed a healed peritoneal site with a thin layer of mesothelial cells on thick scar tissue composed of inflammatory cells and unorganized collagen fibers (Fig. 2b). At day 12, the cecal muscular layer was completely fused to the abdominal wall and the adhesion tissue was less dense and structured compared with the control.

In contrast, defected abdominal walls treated with TB77-100-77 were completely re-epithelialized at days 5 and 12, and showed a continuous layer of mesothelial cells on the inner-layer of the abdominal wall with a thin layer of scar tissue. Macroscopic images showed the smooth-healed abdominal surface and complete absence of adhesions. Debris of TB77-100-77 were still present in the abdominal cavity, suggesting an adequate degradation rate.

**Fig. 3:**
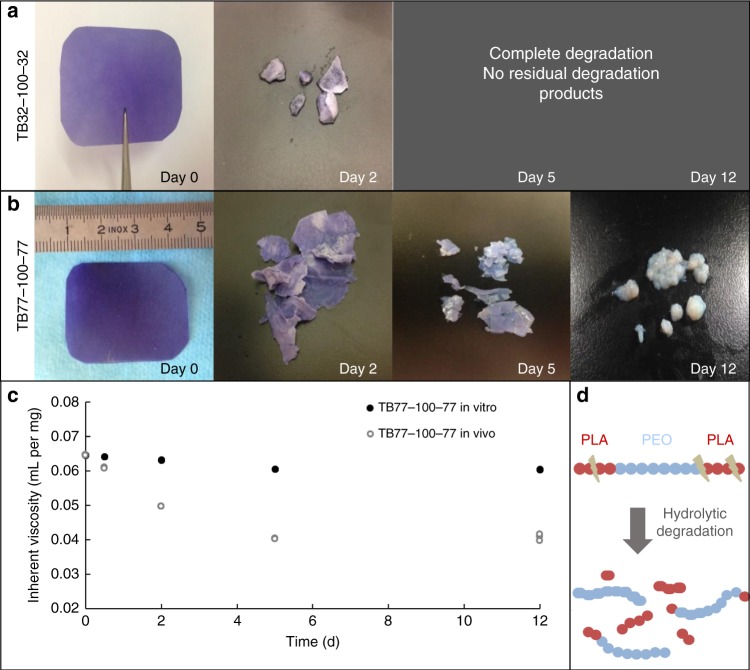
In vivo degradation study of copolymer films after surgical trauma in the abdominal cavity. **a** TB32-100-32 films residues at initial time and after 2, 5, and 12 days. **b** TB77-100-77 films residues at initial time and after 2, 5, and 12 days. **c** Comparison of inherent viscosity of TB77-100-77 films during in vitro degradation after immersion in saline solution and in vivo degradation after surgical trauma. **d** Schematic degradation of triblock copolymers

*Degradation of copolymers:* In vivo degradation of the triblock films in the abdominal cavity was measured through collection of TB32-100-32 and TB77-100-77 residues at 2, 5, and 12 days. No fragments of TB32-100-32 were found after 2 days, indicating complete degradation (Fig. 3a). In contrast, TB77-100-77 was intact at day 2 and residual degradation products were collected at day 12 (Fig. 3b), highlighting the optimal degradation rate of TB77-100-77 compared with TB32-100-32.

We compared in vivo and in vitro degradation of TB77-100-77 by evaluating inherent viscosity values (Fig. 3c), to measure differences in degradation rates. The decrease of viscosity values was more pronounced in vivo compared with in vitro. TB77-100-77 inherent viscosity values at 2 days in vivo were similar to values obtained at 12 days in vitro.

Hydrolytic degradation of PLA-PEO-PLA copolymers in vivo and in vitro was owing to the hydrophilic nature of the PEO block. Water attacks the ester bonds of PLA blocks, resulting in shorter PLA chains and water-soluble fragments (Fig. 3d). PEO ether bonds cannot be cleaved through hydrolytic degradation and PEO blocks become water soluble after degradation of PLA blocks.

Rapid in vivo degradation could be explained by the inflammatory response caused by surgical trauma. In vivo degradation of aliphatic polyesters^34^ are amplified by esterolytic enzymes and reactive oxygen species secreted by macrophages.

TB77-100-77 in vivo degradation extended over 5–6 days and resulted in a physical anti-adhesion barrier, preventing the joining of conjunctive tissues. The estimated time required for complete uterine re-epithelialization is 5–6 days^21,22^. TB77-100-77 was, therefore, chosen for further testing in a postoperative intrauterine adhesion model. The objective was to examine the effectiveness of TB77-100-77 to prevent the joining of conjunctive tissues and promote re-epithelialization.

### In vivo uterine anti-adhesion efficacy of optimal copolymer

We evaluated the anti-adhesion efficacy of TB77-100-77 in an intrauterine adhesion rat model compared with a surgical control group, and a Hyalobarrier® group.

Anti-adherence efficacy was studied in an intrauterine adhesion model developed by Kuramoto et al.^35^, which entailed removing the epithelial layer of uterine horns.

**Fig. 4:**
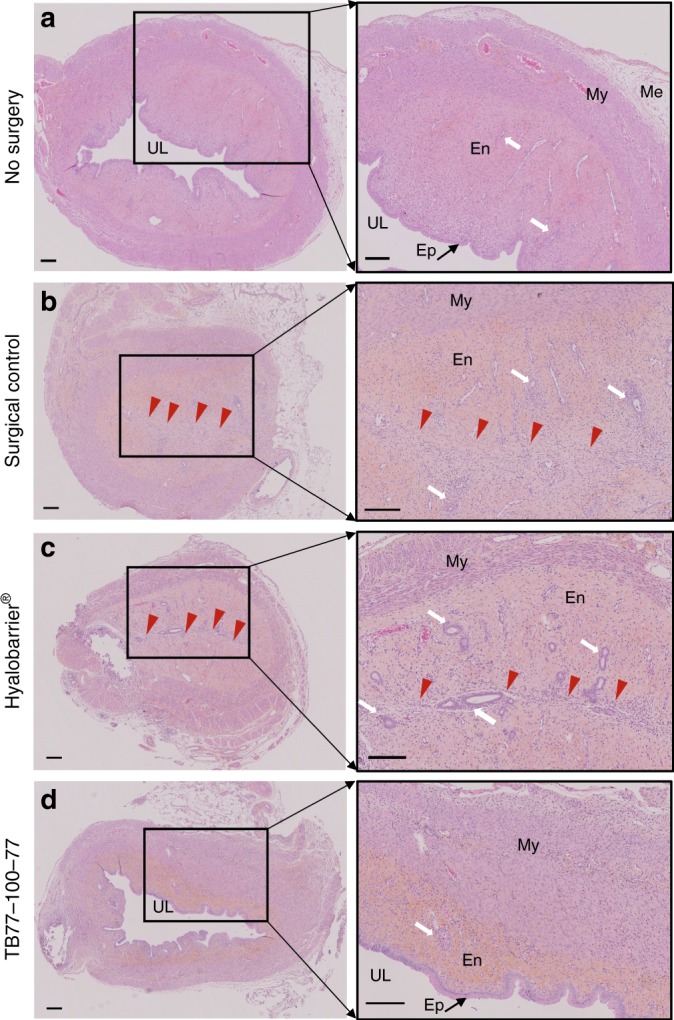
Prevention of intrauterine adhesions. Histological observations and higher magnification of each images of uterine tissues by H&E staining. **a** Normal uterine tissue (no surgery). **b** Intrauterine adhesion 7 days after scraping surgery of the control group. **c** Intrauterine adhesion 7 days after scraping surgery of the Hyalobarrier®-treated group. **d** Uterine tissue 7 days after scraping surgery of the TB77-100-77-treated group. Scale bars in microphotographs indicate 200 µm. UL: uterine lumen; Ep: epithelium; En: endometrium; My: myometrium; Me: mesothelium. Red arrows indicate adhesions

Without surgery (Fig. 4a), uterine histology had a smooth and usual folded structure. Normal uterine tissue (Fig. 4a) showed epithelial cells on the ad luminal surface with a continuous epithelial layer and endometrial glands in the endometrial stromal layer.

In the surgical control, 7 days after scraping, all the horns were obliterated with uterine adhesions, confirming the model’s effectiveness and reproducibility. Histological sections of horns showed that stromal layers adhered to each other, no epithelial cells in the center, and the uterine lumen had disappeared (Fig. 4b). Collagen stained with Red Sirius showed neo-synthesized fibers in the majority of the endometrium, suggesting that the healing process continued after 7 days.

In the Hyalobarrier group (Fig. 4c), histological staining showed an extended inflammatory condition on the endometrial layer with severe stromal adhesions, suggesting that a majority of the endometrium was still in the healing process^36,37^. Small luminal structures with few epithelial cells were observed with no continuous and homogenous luminal structure. Stromal layers adhered to each other in six out of the eight horns treated with Hyalobarrier. No statistical differences were found in adhesion formation incidence between the Hyalobarrier group and the surgical control (*p* = 0.46).

In the TB77-100-77 group, a luminal structure and stratified squamous epithelial layer was visible (Fig. 4d). Collagen staining was localized in the internal part of the endometrium near the epithelial layer, suggesting that the healing process was more complete compared with the other groups. Fibrosis in the TB77-100-77 group was limited to a small section of the endometrium compared with the other groups. Only one out of eight horns in the TB77-100-77 group developed stromal adhesions—a statistically significant reduction in incidence compared with the surgical control (*p* < 0.05).

These results strongly suggest that TB77-100-77 can prevent adhesions in a reproducible rat model of IUAs. The significant decrease of adhesions could be explained by the combination of the anti-adhesion effect of PEO with the degradation rate (> 5 days) of the PLA blocks. The use of an anti-adhesion physical barrier during the reepithelilization process prevented the joining of endometrial walls and uterine adhesion formation. A future study will be conducted to evaluate the effects of TB77-100-77 on spontaneous fertility in rat models who will undergo repeated intrauterine surgery with induced experimental adhesions.

### Ex vivo in utero deployment of optimal copolymer prototype

The following study was conducted to evaluate the clinical usability of TB77-100-77 as an anti-adhesion barrier. Commercial anti-adhesion barriers are introduced by vaginal way after hysteroscopy. Hysteroscopes range in diameter from 2.7 to 10 mm. For TB77-100-77, we used an inserter with a diameter of 5 mm, which was considered ideal for clinical use since it allows the introduction of the larger surface of the anti-adhesion film.

**Fig. 5:**
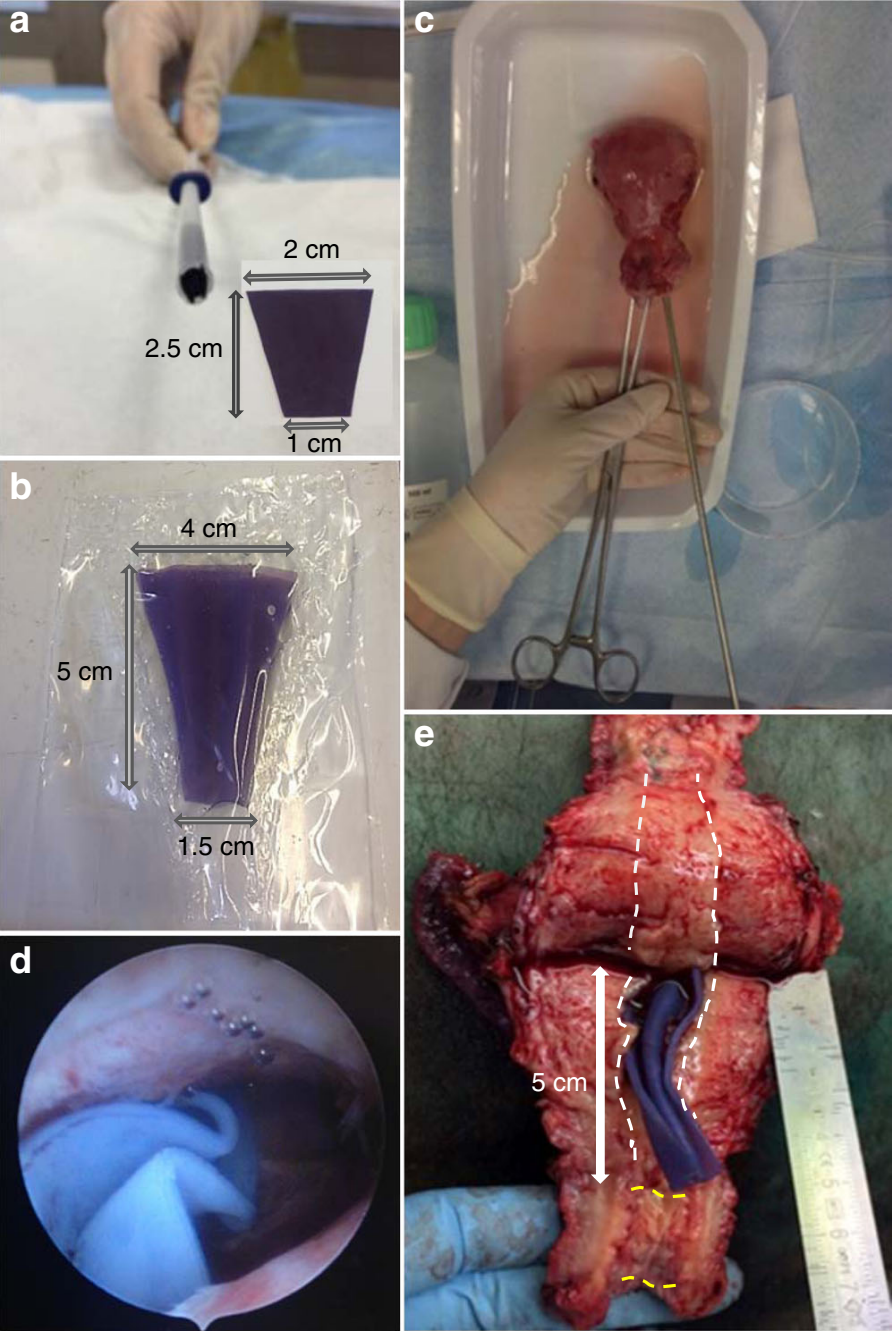
Ex vivo in utero deployment study of TB77-100-77 prototype. **a** TB77-100-77 film folded and introduced into a 5-mm IUD applicator tube; **b** In vitro deployment of the TB77-100-77 film in an anatomical uterus model after 60 min; **c** Illustrations of instillation of physiological saline solution at 37 °C into a uterine cavity. **d** Observations of the prototype deployment by hysteroscopy after 15 min; **e** Opening uterus after 60 min and visualization of the prototype deployment. White dotted lines indicate the delimitation of the uterine cavity and yellow dotted lines indicate the delimitation of the canal cervix

TB77-100-77 films with different trapezoidal morphologies and dimensions were tested to select the prototype with optimal deployment in the uterus. A trapezoidal prototype film of 2 cm at the large base, 1 cm at the short base, 2.5 cm in height and 600 µm in thickness was folded as an accordion and introduced in an IUD applicator 5 mm in diameter (Fig. 5a). The film was pushed into a homemade in vitro uterus model containing physiological serum. An optimal deployment of the film was observed after 60 min. Owing to the serum, the film swelled increasing its surface area, which completely covered the walls of the cavity (Fig. 5b).

An ex vivo in utero deployment was performed in a uterus of a multiparous patient. After a hysterectomy, a physiological saline solution was instilled through the cervix (Fig. 5c) and the prototype in a 5 mm IUD applicator was pushed into the uterine cavity and allowed to expand.

After 15 min, an hysteroscopy was used to observe the swelled film and the volume of uterus (Fig. 5d).

After 60 min, the uterus was transversely cut in order to visualize the deployment of TB77-100-77 (Fig. 5e). The film deployed optimally as it covered the uterine walls from the fundus to the cervix, and its flexibility allowed it to adapt to the uterine morphology. In the future, the film dimensions will have to be determined according to all uterine volumes and the anteriority of patients’ pregnancies (nulliparous and multiparous).

To develop an inserter suitable for intrauterine surgical procedures, it is essential that the tube in which the film is folded should have a diameter in line with the anatomy of the cervix. The design of the inserter should also include ease of use in terms of ergonomics and procedure time.

## Discussion

IUAs lead to recurrent health problems in women that can be debilitating and hinder their ability to bear children. The design of our anti-adhesion intrauterine device took into account usability under surgical conditions and the time needed for complete reepithelization. The device was developed using copolymers of polylactides and high molecular mass PEOs. Two batches of PLA-PEO-PLA with different LA/EO ratios were synthesized and characterized and anti-adhesion, degradation, and swelling properties were evaluated in vitro in order to evaluate the effectiveness of using a high molecular mass PEO. A rat model of sidewall defect–cecum abrasion was used to show the anti-adhesion efficacy and to highlight the necessity to find a copolymer with a degradation time > 5 days. The intrauterine anti-adhesion efficacy of the copolymer (TB77-100-77) that yielded the most promising results was tested in a rat model of postoperative intrauterine adhesions after horn endometrial curettages and showed a decrease of the incidence of adhesions in comparison with a commercial anti-adhesion gel (Hyalobarrier®).

Our prototype was easily inserted via vaginal route in an ex vivo human uterus, quickly deployed within the uterus and showed a capacity to adapt to the uterine morphology by spreading over the entire uterine wall. These results highlighted the interest of using a copolymer with flexible mechanical and swelling properties in an aqueous medium.

The efficacy of the TB77-100-77 copolymer and its comparative advantage over Hyalobarrier®, along with its clinical usability, makes it a promising biomaterial for developing a new medical device that could prevent the formation of postoperative IUAs.

We used a reproducible and relevant model of intrauterine adhesions, which allowed a thorough evaluation of the anti-adhesion effects of the copolymer.

Though we were able to obtain viable results in the small animal model we used in our experiments, we would require a larger study to evaluate the clinical relevance of our prototype.

This study provided important preliminary data for the development of an anti-adhesion intrauterine device that could prevent IUAs and improve rates of fertility. Protocol development of clinical trials for this device is currently underway. The main objective of the clinical trials will be to evaluate the clinical anti-adhesion effect after iatrogenic surgical procedures that lead to infertility.

## Methods

### Materials

PEO (POLYOX™, Mw = 100,000 g mol^−1^) was purchased from Colorcon (Bazainville, France). Tin(II)_2_-ethylhexanoate (Sn(Oct)_2_, 95%), dichloromethane, diethyl ether, tetrahydrofuran, chloroform (CCL_3_) were purchased from Sigma-Aldrich (St-Quentin Fallavier, France). d,l-lactide (d,l-LA) were purchased from Corbion (Gorinchem, The Netherlands). Minimum essential medium (MEM), horse serum, l-glutamine, phosphate-buffered saline, penicillin, streptomycin, amphotericin B, and non-essential amino acid were purchased from Gibco (Saint Aubin, France). Twenty-four-well non adhesive culture plates were purchased from Becton Dickinson (Le Pont de Claix, France).

### Synthesis and characterization of copolymers

Two PLA-PEO-PLA triblocks copolymers (named TB32-100-32 and TB77-100-77) were synthesized by ring-opening polymerization of d,l-lactide from PEO 100,000 g per mol as initiator. Typically, 10 g of PEO and various amounts of d,l-lactide (20 and 10 g) were introduced into two flasks, the initial molar ratio of lactic acid to ethylene oxide LA/EO were 1.2 (TB77-100-77) and 0.6 (TB32-100-32). PEO and d,l-lactide were vacuum dried for 24 h. Tin (II)−2 éthylhexanoate (0.1% of the number of hydroxyl functions of PEO) were added in dried polymerization flasks. After degassing, the flask was sealed under vacuum and polymerization was carried out at 130 °C for 5 days. Poly(d,l-lactide) (named PDLLA) was synthesized as described by Garric et al. in 2005 and 2008^20,38^. In brief, freshly recrystallized d,l-lactide was mixed with Tin (II)-2 éthylhexanoate in a 100 mL round bottom flask. The flask was sealed under dynamic vacuum after degassing through vacuum/argon cycles. The feed was allowed to polymerize at 130 °C for 5 days. The resulting material was dissolved in acetone and the solution was filtered through a 5G sintered-glass filter. The PDLLA polymer was precipitated by addition of methanol and then washed several times with small amounts of methanol. The recovered compound was allowed to dry under vacuum for 10 days at 40 °C.

The composition was evaluated using nuclear magnetic resonance spectroscopy (^1^H-NMR spectroscopy) at room temperature with an AMX300 Brucker spectrometer (300 MHz), using CDCl_3_ was used as a solvent and trimethylsilane as the internal standard.

The inherent viscosity (dl per g) was measured by capillary viscometer (Schott Ubbelohde; Schott Instruments GmbH, Mainz, Germany) at 25 °C using a CHCl_3_ solvent.

The glass transition (Tg) and melting temperature (Tm) were studied using Differential Scanning Calorimetry (Perkin-Elmer DSC6000).

The number average molecular mass of PDLLA and PEO were determined by SEC using a Viscotek GPC Max autosampler and a Viscotek RI detector. Molecular mass was expressed according to polystyrene standards and with tetrahydrofuran as a mobile phase for PDLLA and PEO standards, with water as a mobile phase for PEO.

### In vitro evaluation of copolymers

*Swelling properties:* Water uptake was conducted to evaluate swelling properties (Fig. 1a). Triblocks and PDLLA films were created using a solvent casting method. A total of 1.6 g of copolymer powder was solubilized in 15 mL of dichloromethane. The solution was poured into an aluminum plate and the solvent was evaporated under vacuum for 48 h.

In order to determine the water content versus incubated time, polymer films were incubated in a phosphate-buffered saline (pH 7.4) at 37 °C under agitation

Filter paper was used to remove surface water. Films were weighted and the percent of water uptake was calculated as follows:

$${\mathrm{Water}}\,{\mathrm{uptake}}\left( \% \right) = \left( {{\mathit{m}}_{\mathit{t}} - {\mathit{m}}_0} \right) \ast 100{\mathit{/m}}_0$$

where *m*_*t*_ is the weight measured at *t* time, and *m*_0_ is the weight of the dry sample measured at *t* = 0 h.

*Degradation of copolymers:* Triblocks and PDLLA films were incubated in phosphate-buffered saline (pH 7.4) at 37 °C. The samples were lyophilized and the weight loss calculated.

The inherent viscosity was measured by capillary viscometer (Schott Ubbelohde; Schott Instruments GmbH, Mainz, Germany) at 25 °C using CHCl_3_ as a solvent (Fig. 1b). The capillary number was 532 03/0c with a diameter of 0.46 mm and the polymer concentration was 5 mg/mL.

*Cell adhesion effect:* A comparative evaluation of cell anti-adhesion properties was conducted on the triblock copolymer films (TB32-100-32 and TB77-100-77), PDLLA film, Seprafilm® (an anti-adhesion barrier currently in use), and the control TCPS). The data of this comparative evaluation is provided in Fig. 1c. NCTC-Clone 929 is recommended for the evaluation of in vitro cytocompatibility of medical devices according to EN ISO 10993-5. In this study, NCTC-Clone 929 cells (mouse fibroblast cell line (ECACC 85011425)) were cultured in 500 ml of MEM with 5 mL of glutamax (1% stabilized glutamine), 50 mL of horse serum, and 100 U per mL penicillin and streptomycin 100 μg per mL.

PrestoBlue® assay was used to evaluate cell adhesion. Film samples were sterilized by gamma irradiation (Synergy Health Company, Marseille, France). The films were treated with an irradiation dose between 29.8 and 33.8 KGy and then stored at – 20 °C before being placed in a 24-well plate in triplicate. In total of 150,000 cells were seeded directly onto polymer films, Seprafilm® and directly in the well of TCPS. Each well was maintained in the incubator for 45 min, 1 h 30 min, and 3 H. A total of 500 μL of a 10% solution of PrestoBlue® was introduced into each well and incubated for 1 H. In total, 200 μL of the solution were taken from each well and placed into a 96-well plate.

Fluorescence readings were measured using an excitation of 530 nm and an emission of 615 nm using a spectrophotometer multi-plate reader (VictorX3 multi-label plate reader, Perkin-Elmer).

### In vivo evaluation of copolymers

We first evaluated the ideal degradation time for complete uterine re-epithelialization to allow for optimal anti-adhesion effect and selected the optimal copolymer.

Experiments were approved by the Ethics Committee of the French Ministry of Education and Research (contract number 02367.01, task order 1065) and conducted according to the Guide for the Care and Use of Laboratory Animals. All efforts were made to minimize animal suffering and to use the minimum number of animals necessary to produce reliable scientific data. They were housed in individual cages in a room at 22 °C with a humidity rate of 55% (± 10%) with free access to food (SAFE®) and tap water. They were examined, weighed, and their litter changed daily, respecting the guide of good practices and animal welfare.

*Anti-adhesion effect in peritoneal adhesion prevention*: The colored copolymer films were made using the solvent casting method. In total, 1.6 g of copolymer powder was solubilized in 15 mL of dichloromethane in 1 mg of water-insoluble violet dye (Dye Reference: D&C Violet 2 K7014 Sensient Technologies). The solution was poured into an aluminum plate and the solvent was evaporated for 48 h. The copolymer films were sterilized by gamma irradiation (Synergy Health Company, Marseille, France), treated with an irradiation dose between 29.8 kGy and 33.8 KGy and then stored at –20 °C until the day of surgery.

A total of 48 Wistar females (250/275 g and 8–10 weeks, provided by Charles River Laboratories) were anesthetized with intramuscular injections of ketamine (50 mg/kg) and xylazine (5 mg/kg). The abdomen was disinfected with Betadine® and a local subcutaneous anesthesia (Lidocaine 0.1%) was performed before the incision. A median vertical incision of 4 cm long in the abdomen was made and the abdominal cavity was opened. The cecum was abraded with a compress until a punctiform hemorrhage and muscle of the abdominal wall was excised (1 cm^2^).

The randomized study consisted of four groups of 12 female rats: implantation of sterile film (TB32-100-32); implantation of sterile film (TB77-100-77), instillation of Hyalobarrier®, and no treatment (control group). Each group of 12 was evaluated at day 5 and day 12 with six rats for each day.

Copolymer films were cut into a rectangle of 4 cm long and 3.5 cm wide. The cut films were placed directly in the flank between the cecum and the peritoneum. One microliter of physiological saline was instilled into the abdominal cavity before closure. In the Hyalobarrier® group, 2 mL of Hyalobarrier® gel was instilled between the cecum and the abdominal wall. For the control group (surgical control), the abdomen was sutured directly after cecum abrasion and excision of the peritoneum. Buprenorphine (0.02 mg/kg) was systematically administered within 48 h of the surgical procedure.

Six rats were killed at day 5 and 6 at day 12. The adhesion score (Table 2) was evaluated as a function of the extent, severity of the adhesions and the traction necessary to free the adherent organs (degree of adhesions)^39^. The animals were killed by ketamine anesthesia and lethal injection of pentobarbital.

Samples of adhesions resulting from the cecum, the abdominal wall and the surrounding tissues were immersed in 10% formalin at room temperature for 24 h, washed twice in phosphate-buffered saline and then immersed in 70% ethanol. Samples were embedded in paraffin. Paraffin wax blocks were cut into 3 µm-thick sections. Prepared sections were then stained with hematoxylin–eosin and sirius red. An anatomopathologist, blinded to the origin of the samples, examined the tissues to conduct the scoring of adhesions (Fig. 2).

*Degradation of copolymers:* For this study, residual copolymers from the rats that were euthanized at 5 and 12 days (in the randomized anti-adhesion study described above) were used to evaluate the degradation of the copolymers at 5–12 days. To evaluate the degradation of the copolymers at 2 days, three additional rats that underwent the same surgical procedure described the anti-adhesion study. The three rats were euthanized after 2 days of surgery to determine whether adhesions were formed.

Residues of copolymers were lyophilized, weighed, and the inherent viscosity was measured by capillary viscometer.

### In vivo uterine anti-adhesion efficacy of optimal copolymer

Twelve Wistar females (250/275 g and 8–10 weeks) were anesthetized after intramuscular injection of ketamine (50 mg per kg) and xylazine (5 mg per kg). The abdomen was disinfected with Betadine® and local subcutaneous anesthesia was performed before incision (Lidocaine 0.1%). A 4 cm in length vertical median incision of the abdomen was made and the abdominal cavity was opened. Both of the horns in each female were incised along their length (about 2 cm) and the inner wall was abraded with a knife blade. In total, there were 24 horns that were distributed into three groups of 8 horns and we ensured that the 2 horns of each rat were placed in different groups (group 1 had no treatment, group 2 were instilled with Hyalobarrier® and in group 3 sterile copolymer filament (TB77-100-77 S) were implanted). Hyalobarrier® gel and a copolymer filament were placed in incised horns and the horns were sutured with resorbable sutures (5/0 Vicryl).

An additional rat did not undergo the surgical procedure was used as a control reference to show a normal uterine histology (Fig. 4a No Surgery).

Filaments with a diameter of 1.5 mm were produced by hot extrusion with a Noztek Powder Filament Extruder (Shoreham, England). Filaments were sterilized by gamma irradiation (Synergy Health society) with an irradiation dose between 27.5 and 30.6 kGy and stored at −20 °C until the day of surgery.

Uterine horns of the 3 groups and the non-surgical female rat used as a control were collected and embedded in paraffin. Paraffin wax blocks were cut into 3 µm-thick sections. Prepared sections were then stained with hematoxylin–eosin and Sirius red. An anatomopathologist, blinded to the origin of the samples, examined and conducted adhesion scoring for the collected horns.

Next we evaluated the clinical usability for gynecological surgery.

### Ex vivo in utero deployment of the optimal copolymer prototype

The prototype was made from a film of TB77-100-77 obtained by using the same protocol as described before and cut to form a trapezoid with a large base of 2 cm, and a short base of 1 cm, a height of 2.5 cm and a thickness of 600 µm. The film was folded so that it could be introduced in an IUD inserter tube of 5 mm in diameter. The prototype deployment study was first conducted in a homemade in vitro anatomical uterus model. The anatomical model was designed from two polyethylene films welded together using a heated spatula. The dimensions of the model (4 cm at the top of the uterus; 1.5 cm at the base of the uterus; 5 cm along the length of the uterus) were chosen to closely match those of a uterus of a multiparous patient.

The uterus used in this study was obtained from a patient who underwent a hysterectomy at the gynecology and obstetrics department of the University Hospital of Nîmes in France. This study was approved by the Ethics Review Committee on Human Research of University Hospital of Nîmes (IRB no. 105604) and an informed consent was obtained from a multiparous patient (33 years old) who underwent a laparoscopic hysterectomy surgery for adenomyoisis. The uterus was placed in an incubator at 37 °C. Hysteroscope was introduced via the cervix to obtain a clear view of the uterine cavity and the absence of uterine malformation, fibroids or abnormal tumors or tissues were recorded. Physiological saline solution was instilled via the cervix with the hysteroscope.

After pushing the TB77-100-77 film into the uterine cavity containing physiological saline solution, a hysteroscope was used to observe the swelling and deployment properties of the copolymer film after 15 min. After 60 min, the uterus was transversely cut in order to observe the deployment of the prototype in the uterine cavity.

### Statistics and reproducibility

*In vitro cell adhesion evaluation:* Data are presented as mean ±standard deviations. Statistics were derived from three separate samples. Statistical analysis was conducted using a Kruskal–Wallis test and a Holm’s method to make a comparison between the polymers and control (Seprafilm®, PDLLA, and TCPS).

*Anti-adhesion effect in peritoneal adhesions prevention*: All quantitative values are expressed as mean ± SD. R statistical software was used to perform statistical analysis. We estimated the number of animal to demonstrate a reduction of adhesion score of 10/10 in control group to 5/10 in the TB77-100-77 group. The number of animal was six animals in each group to achieve an alpha risk at 5% and power at 80%. Kruskall–Wallis test was used to compare adhesion scores. The pairwise group comparisons were made by Mann–Whitney *U* test. Test of significance were two-sided with a 0.05 alpha risk.

### Reporting summary

Further information on research design is available in the Nature Research Reporting Summary linked to this article.

## Supplementary information

Reporting Summary

Description of Additional Supplementary Files

Supplementary Data 1

## Data Availability

Raw data used for Fig. 1 and Fig. 3 are available in Supplementary Data 1. All other data are available from the corresponding author upon reasonable request.
