# Supplementary material for: Preliminary design of a new degradable medical device to prevent the formation and recurrence of intrauterine adhesions
Source: Commun Biol. 2019 May 22;2:196. doi: 10.1038/s42003-019-0447-x (PMC6531438; doi:10.1038/s42003-019-0447-x)
Supplement: Supplementary file 1 — Reporting Summary [file 42003_2019_447_MOESM1_ESM.pdf]

nature research

Corresponding author(s): Garric Xavier
Last updated by author(s): Apr 12, 2019

Reporting Summary

Nature Research wishes to improve the reproducibility of the work that we publish. This form provides structure for consistency and transparency in reporting. For further information on Nature Research policies, see Authors & Referees and the Editorial Policy Checklist.

Statistics

For all statistical analyses, confirm that the following items are present in the figure legend, table legend, main text, or Methods section.

n/a	Confirmed	
☐	☒	The exact sample size (*n*) for each experimental group/condition, given as a discrete number and unit of measurement
☐	☒	A statement on whether measurements were taken from distinct samples or whether the same sample was measured repeatedly
☐	☒	The statistical test(s) used AND whether they are one- or two-sided<br>*Only common tests should be described solely by name; describe more complex techniques in the Methods section.*
☒	☐	A description of all covariates tested
☒	☐	A description of any assumptions or corrections, such as tests of normality and adjustment for multiple comparisons
☐	☒	A full description of the statistical parameters including central tendency (e.g. means) or other basic estimates (e.g. regression coefficient) AND variation (e.g. standard deviation) or associated estimates of uncertainty (e.g. confidence intervals)
☐	☒	For null hypothesis testing, the test statistic (e.g. *F*, *t*, *r*) with confidence intervals, effect sizes, degrees of freedom and *P* value noted<br>*Give P values as exact values whenever suitable.*
☒	☐	For Bayesian analysis, information on the choice of priors and Markov chain Monte Carlo settings
☒	☐	For hierarchical and complex designs, identification of the appropriate level for tests and full reporting of outcomes
☒	☐	Estimates of effect sizes (e.g. Cohen's *d*, Pearson's *r*), indicating how they were calculated

Our web collection on statistics for biologists contains articles on many of the points above.

Software and code

Policy information about availability of computer code

Data collection	No software was used during data collection
Data analysis	Data analyses were performed using the statistical software "R" (R Foundation for Statistical Computing, R Development Core Team, 2004).

For manuscripts utilizing custom algorithms or software that are central to the research but not yet described in published literature, software must be made available to editors/reviewers. We strongly encourage code deposition in a community repository (e.g. GitHub). See the Nature Research guidelines for submitting code & software for further information.

Data

Policy information about availability of data

All manuscripts must include a data availability statement. This statement should provide the following information, where applicable:

- Accession codes, unique identifiers, or web links for publicly available datasets
- A list of figures that have associated raw data
- A description of any restrictions on data availability

Raw data used for Figures 1 and 3 are available in Supplementary Data 1. All other data may be available from the corresponding author upon reasonable request.

Field-specific reporting

Please select the one below that is the best fit for your research. If you are not sure, read the appropriate sections before making your selection.

☒ Life sciences ☐ Behavioural & social sciences ☐ Ecological, evolutionary & environmental sciences

Life sciences study design

All studies must disclose on these points even when the disclosure is negative.

Sample size	Sample sizes were chosen based on pilot experiments in order to provide sufficient power for statistical comparisons. We estimated the number of animals to demonstrate a reduction of adhesion score of 10/10 in control group to 5/10 in the TB77-100-77 group. The number of animals chosen was 6 in each group to achieve an alpha risk of 5 % and power at 80%.
Data exclusions	No data were excluded from the analyses.
Replication	The physico-chemical and cell biology experiments in this paper have been repeated twice (for a total of 3 independent experiments). We did not observe any failures of the replication experiments.
Randomization	The randomized study consisted of four groups of 12 female rats: implantation of sterile film (TB32-100-32); implantation of sterile film (TB77-100-77), instillation of Hyalobarrier® (HB) and no treatment (control group). Each group of 12 was evaluated at day 5 and day 12 with 6 rats for each day.
Blinding	An anatomopathologist, blinded to the origin of the samples, examined the tissues to conduct the scoring of adhesions

Reporting for specific materials, systems and methods

We require information from authors about some types of materials, experimental systems and methods used in many studies. Here, indicate whether each material, system or method listed is relevant to your study. If you are not sure if a list item applies to your research, read the appropriate section before selecting a response.

Materials & experimental systems

n/a	Involved in the study	
☒	☐	Antibodies
☐	☒	Eukaryotic cell lines
☒	☐	Palaeontology
☐	☒	Animals and other organisms
☐	☒	Human research participants
☒	☐	Clinical data

Methods

n/a	Involved in the study	
☒	☐	ChIP-seq
☒	☐	Flow cytometry
☒	☐	MRI-based neuroimaging

Eukaryotic cell lines

Policy information about cell lines

Cell line source(s)	NCTC-Clone 929 cells (L929) from ECACC (European Collection of Authenticated cell cultures); reference: 85011425
Authentication	Batch number: 13l043
Mycoplasma contamination	NCTC-Clone 929 cells were tested negative for mycoplasma contamination (test: LONZA LT07-218; batch: 5799444)
Commonly misidentified lines (See ICLAC register)	No commonly misidentified cell lines were used in this study

Animals and other organisms

Policy information about studies involving animals; ARRIVE guidelines recommended for reporting animal research

Laboratory animals	Wistar rats : Female (250/275 g and 6-8 weeks) from Charles River Laboratories
Wild animals	n/a
Field-collected samples	n/a
Ethics oversight	Experiments were approved by the Ethics Committee of the French Ministry of Education and Research (contract number 02367.01, task order 1065) and conducted according to the Guide for the Care and Use of Laboratory Animals. All efforts were made to minimize animal suffering and to use the minimum number of animals necessary to produce reliable scientific data.

Note that full information on the approval of the study protocol must also be provided in the manuscript.

Human research participants

Policy information about studies involving human research participants

Population characteristics	The uterus used in this study was obtained from a patient who underwent a hysterectomy at the Gynecology and Obstetrics Department of the University Hospital of Nîmes in France. The multiparous patient (33 years old) underwent a laparoscopic hysterectomy surgery for adenomyoisis.
Recruitment	The criteria used for selection was that the patient needed to be at least 18 years of age, underwent a Laparoscopic hysteroctomy for a benign pathology (adenomyosis, Myofibromatous uterus without impact on the uterine cavity, unexplained menorrhagia without risk of malignancy).
Ethics oversight	This study was approved by the Ethics Review Committee on Human Research of the University Hospital of Nîmes (IRB n°105604) and an informed consent was obtained from the patient.

Note that full information on the approval of the study protocol must also be provided in the manuscript.
