# Supplementary material for: Preliminary design of a new degradable medical device to prevent the formation and recurrence of intrauterine adhesions
Source: Commun Biol. 2019 May 22;2:196. doi: 10.1038/s42003-019-0447-x (PMC6531438; doi:10.1038/s42003-019-0447-x)
Supplement: Supplementary file 2 — Description of Additional Supplementary Files [file 42003_2019_447_MOESM2_ESM.pdf]

Description of Additional Supplementary Files

File Name: Supplementary Data 1
Description: This file contains all data presented in the figures 1 and 3.
